# FETR-ALS Study Protocol: A Randomized Clinical Trial of Fecal Microbiota Transplantation in Amyotrophic Lateral Sclerosis

**DOI:** 10.3389/fneur.2019.01021

**Published:** 2019-09-20

**Authors:** Jessica Mandrioli, Amedeo Amedei, Giovanni Cammarota, Elena Niccolai, Elisabetta Zucchi, Roberto D'Amico, Federica Ricci, Gianluca Quaranta, Teresa Spanu, Luca Masucci

**Affiliations:** ^1^Neurology Unit, Department of Neuroscience, S. Agostino Estense Hospital, Azienda Ospedaliero Universitaria di Modena, Modena, Italy; ^2^Laboratory of Immunology, Department of Experimental and Clinical Medicine, University of Florence, Florence, Italy; ^3^Scienze Gastroenterologiche, Endocrino-Metaboliche e Nefro-Urologiche, Fondazione Policlinico Universitario A. Gemelli IRCCS, Università Cattolica del Sacro Cuore, Rome, Italy; ^4^Istituto di Medicina Interna e Geriatria, Università Cattolica del Sacro Cuore, Rome, Italy; ^5^Department of Biomedical, Metabolic and Neural Sciences, University of Modena and Reggio Emilia, Modena, Italy; ^6^Statistics Unit, Department of Medical and Surgical Sciences for Children & Adults, University of Modena and Reggio Emilia, Modena, Italy; ^7^Instituto di Microbiologia, Università Cattolica del Sacro Cuore, Rome, Italy; ^8^Dipartimento di Scienze di Laboratorio ed Infettivologiche, Fondazione Policlinico Universitario A. Gemelli IRCCS, Rome, Italy

**Keywords:** amyotrophic lateral sclerosis, microbiota, adaptive immunity, fecal microbiota transplantation, T cells, treg lymphocytes, randomized controlled clinical trial

## Abstract

**Background and Rationale:** Among the key players in the pathogenesis of Amyotrophic Lateral Sclerosis (ALS), microglia and T regulatory lymphocytes (Treg) are candidate cells for modifying the course of the disease. The gut microbiota (GM) acts by shaping immune tolerance and regulating the Treg number and suppressive function, besides circulating neuropeptides, and other immune cells that play in concert through the gut-brain axis. Previous mouse models have shown an altered enteric flora in early stage ALS, pointing to a possible GM role in ALS pathogenesis. Fecal Microbial Transplantation (FMT) is a well-known therapeutic intervention used to re-establish the proper microenvironment and to modulate enteric and systemic immunity.

**Methods:** We are going to perform a multicenter randomized double-blind clinical trial employing FMT as a therapeutic intervention for ALS patients (NCT0376632). Forty-two ALS patients, at an early stage, will be enrolled with a 2:1 allocation ratio (28 FMT-treated patients vs. 14 controls). Study duration will be 12 months per patient. Three endoscopic procedures for intestinal biopsies in FMT and control groups are predicted at baseline, month 6 and month 12; at baseline and at month 6 fresh feces from healthy donors will be infused at patients in the intervention arm. The primary outcome is a significant change in Treg number between FMT-treated patients and control arm from baseline to month 6. Secondary outcomes include specific biological aims, involving in-depth analysis of immune cells and inflammatory status changes, central and peripheral biomarkers of ALS, besides comprehensive analysis of the gut, saliva and fecal microbiota. Other secondary aims include validated clinical outcomes of ALS (survival, forced vital capacity, and modifications in ALSFRS-R), besides safety and quality of life.

**Expected Results:** We await FMT to increase Treg number and suppressive functionality, switching the immune system surrounding motorneurons to an anti-inflammatory, neuroprotective status. Extensive analysis on immune cell populations, cytokines levels, and microbiota (gut, fecal and saliva) will shed light on early processes possibly leading the degenerative ALS course.

**Conclusions:** This is the first trial with FMT as a potential intervention to modify immunological response to ALS and disease progression at an early stage.

## Introduction

When questioning why so many failures in clinical trials in amyotrophic lateral sclerosis (ALS), the lack of a complete comprehension of the pathogenic systems behind the disease onset and progression might be accounted as one of the main reasons. Indeed, ALS is a complex syndrome. Aberrant cellular pathways convey from protein misfolding, with endoplasmic reticulum stress, defective autophagy and damage to cytoskeleton (1), associated to staggered RNA processing and mitochondria homeostasis, increased oxidative stress, enhanced excitotoxicity, reduced neurotrophic sustenance, and to a glial inflammatory response that is oriented toward a harmful side (2).

Recent studies highlighted the role of microglia and opened new perspectives in the knowledge of the non-cell autonomous molecular mechanisms possibly contributing to ALS, launching them as a plausible target for many clinical trials. During ALS progression, activated microglia switch from the M2 phenotype, which is neuroprotective and supports tissue repair and neuron survival through the release of neuroprotective factors, to M1 phenotype, which is toxic and contributes to neuronal death through pro-inflammatory cytokines production, and tissue destruction. Therapeutic approaches targeting microglia polarization to induce the M2 phenotype are promising strategies to contain local neurodegeneration and improve ALS outcome (3). Indeed, in animal models of the disease, diminishing the mutant levels of microglia sharply slowed later disease progression (4).

M1/M2 macrophages phenotypes switch have been shown to be induced by CD4^+^ T cells, especially CD4^+^CD25^+^Foxp3^+^ T regulatory (Treg) cells (5, 6). In the blood and spinal cord of patients with ALS, CD4^+^ T cells (T helper—Th) are increased, especially with a predominantly pro-inflammatory Th1/Th17 phenotype (7). On the contrary, Tregs from blood of ALS patients demonstrated a significant decrease in the ability to suppress the proliferation of the effector T cells; and of note, the loss extent in suppression was correlated with disease progression (7). The passive transfer of mSOD1 Tregs into ALS mice lacking functional T lymphocytes prolonged their survival while FoxP3 mRNA in the spinal cord of mSOD1 mice inversely correlated with disease progression (8). Finally, in ALS patients the Tregs' number and percentage, and FoxP3 expression decreased with faster disease progression and were early predictors of ALS progression and survival (8, 9).

Very recently, autologous infusions of expanded Treg cells and concomitant IL-2 into patients with ALS resulted to be safe and tolerable during early and later stages of disease in a phase I study, where infusions seemed to slow progression rates (10). Moreover, the study detected a correlation between Treg suppressive function and disease progression, underscoring the rationale underlying the use of Treg suppressive functionality as an indicator of clinical status (10).

All these evidences convey toward a dysfunction of the adaptive immune response during ALS. Increasing data suggest that the systemic immune response and especially the neuroimmune system can be modulated by gut microbiota (GM) through the gut-brain axis, a key player in the regulation of mutual signaling between gut microflora and central nervous system (CNS) (11, 12) employing bidirectional communication via neuronal, hormonal, immunologic, and toxic signaling (13–15).

Intestinal microbiota includes a complex ecosystem with an exceptionally high bacterial density and diversity: the adult alimentary tract contains 1–2 kg of microbial cells of hundred bacterial species, of which over 80% have not been cultured (16–18).

GM communicates straightforward with the enteric immune system, shaping immune tolerance and thus contributing to the modulation of immune reactions during inflammation (19). Conversely, upon pathogen invasion, dysbiosis or barrier break, the microbe-associated molecular patterns stimulate macrophages and dendritic cells to produce pro-inflammatory cytokines. In turn, the cytokine activate the adaptive immune cells, thus contributing to the breakdown of immune homeostasis (20), and typically, determines loss of the immune cells that keep the aggressiveness of the immune system in check, namely Tregs (12).

Several brain biological processes may be influenced by GM alterations. Germ free mice have been found to have an altered density, morphology and maturity of microglia, and treatment with a short chain fatty acids (SCFA) mixture restored the density and morphology of CNS immune cells, suggesting that GM can influence both the development and functions of microglia (12, 21).

In ALS, GM dysbiosis may facilitate the disease onset or drive its progression and related outcomes, in the presence of other risk factors. Alternately, the GM dysbiosis may be (further) altered by the disease presence and in some individuals contribute to disease progression, prognosis, also in terms of variable response to drug treatments (22).

An alteration in the intestinal bacterial flora as an external trigger could explain the rare cases of ALS in spouses or in some clusters (23).

Based on these premises, to treat GM dysbiosis through microbiota restoration would have the potential to interfere and slow ALS progression (24).

Our trial aims at evaluating the biological basis of a potential treatment for ALS (namely fecal microbiota transplantation, FMT) in order to plan a following efficacy study.

The primary objective is to evaluate if FMT augments Tregs' number in ALS patients, treated with FMT compared to the control arm and measured at baseline and at month 6.

The secondary objectives include specific biological aims: (i) comparison between treated patients and control arm of Tregs'number and T cell subsets at different time points in blood and gut tissue samples; (ii) comparison between treated patients and control arm of neurophylaments and CSF cytokines and cells; (iii) analysis of fecal and saliva samples between the two groups to evaluate microbiota and cytokines' profile; (iv) FMT safety and tolerability in ALS; (v) clinical assessment (including tracheostomy-free survival, Forced vital capacity score, ALSFRS-R score, frequency of PEG or NIV); finally, (vi) the Quality of Life (QoL) assessment.

## Methods and Analysis

### Study Design

We are going to perform a randomized double blind multi-center study on FMT in ALS.

**Figure 1:**
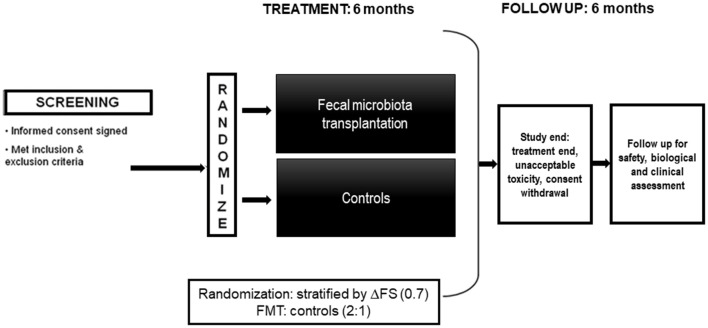
Picture of the study design.

The Figure 1 summarizes the study design.

The study will include 42 ALS patients with 2:1 allocation in 2 groups of subjects (28 FMT vs. 14 controls). Patients will be screened in the 15 days before baseline; then they will be randomly allocated to either FMT or control group. Randomization will be 2:1 (FMT: controls) and will be performed on line (using a computer-generated list of random number that will be centrally generated in the Statistical Unit). Given the heterogeneous ALS progression, patients will be stratified by ΔFS (progression rate), calculated at randomization according to Kimura et al. (25). Riluzole will be maintained during the entire study duration unless adverse events or patients' decision to withdraw. Endoscopic treatment will be performed within 21 days from randomization. Randomization number will not be re-used in any case. Estimated enrolment time is 18 months.

### Study Population

The study will include probable laboratory-supported, clinically probable, or definite ALS according to revised El Escorial criteria (sporadic and familiar). Inclusion and exclusion criteria for the enrolment of the patients are presented in the Table 1.

**Table 1:** Inclusion and exclusion criteria for patients.

**Inclusion criteria**	**Exclusion criteria**
– Patients diagnosed with a laboratory supported, clinically “probable” or “definite” amyotrophic lateral sclerosis according to the Revised El Escorial criteria – Sporadic or familial ALS – Female or male patients aged between 18 and 70 years old – Disease duration from symptoms onset no longer than 18 months at the screening visit – Patients treated with a stable dose of Riluzole (100 mg/days) for at least 30 days prior to screening – Patients with a weight >50 kg and a BMI ≥18 – Patients with a FVC equal or more than 70% predicted normal value for gender, height, and age at the screening visit – Patients able and willing to comply with study procedures as per protocol – Patients able to understand, and capable of providing informed consent at screening visit prior to any protocol-specific procedures – Use of effective contraception both for males and females	– Known organic gastrointestinal disease – History of gastrointestinal malignancy; ongoing malignancies – Use of immunosuppressive or chemotherapy within the past 2 years – Celiac disease and/or food (e.g., lactose) intolerance – Previous gastrointestinal surgery – Any condition that would make endoscopic procedures contraindicated – Acute infections requiring antibiotics – Antimicrobial treatment or probiotics 4 weeks prior to screening – Severe comorbidities (heart, renal, liver failure); severe renal (eGFR < 30 ml/min/1.73 m^2^), or liver failure or liver aminotransferase (ALT/AST > 2x Upper limit of normal), – Autoimmune diseases, inflammatory disorders (SLE, Rheumatoid arthritis, connective tissue disorder) or chronic infections (HIV, hepatitis B, or C infection, Tuberculosis) – Abuse of alcohol or drugs – Participation in clinical trials <30 days before screening – Existing blood dyscrasia (e.g., myelodysplasia) – White blood cells <4,000/mm3, platelets count <100,000/mm3, hematocrit <30% – Patients who underwent non-invasive ventilation, tracheotomy and /or gastrostomy – Women who are pregnant or breastfeeding

*BMI, Body Mass Index; FVC, Forced Vital Capacity; eGFR, estimated Glomerular Filtration Rate; ALT, alanine aminotransferase; AST, aspartate aminotransferase; SLE, Systemic Lupus Erythematosus*.

### Interventional Methods

Treatment will be double blinded to patients and neurologists, but not to the endoscopist and microbiologist. ALS patients will undergo upper GI endoscopy with small-intestine biopsies (*n*° 4 biopsies of small intestine, performed with a standard biopsy forces) at baseline and after 6 and 12 months. At baseline, the patients will be randomized (2:1) to either an allogenic (from donors) infusion of collected feces (60 grams) (FMT) in the duodenum-jejunum or no treatment (control group). The infusion will be performed through a standard nasojejunal tube that will be placed during endoscopy. Fecal infusion will be repeated at month 6. Control group patients will not receive any treatment (at baseline or at month 6), but will remain blind to treatment because of sedation due to small-intestine biopsies. Fecal microbiome will be diluted in saline solution (200 ml) and infused at 30 ml/min speed (whole amount of the performance: 15 min). Fresh feces for the FMT will be obtained by habitual healthy donors for *Clostridium difficile* infection. The Table 2 shows the blood and stool testing for donators and the general selection criteria. Before preparation, sample donation will be always analyzed by rapid molecular test to detect intestinal pathogens.

**Table 2:** Criteria for donors' selection.

**EXCLUSION CRITERIA**		
**Infectious diseases risk**	**Gastrointestinal, metabolic and neurological disorders**	**Drugs that can impair gut microbiota composition**
▸ History of, or known exposure to, HIV, HBV, HCV, syphilis, HTLV1-2, tuberculosis, malaria, trypanosomiasis ▸ Known systemic infection not controlled at the time of donation ▸ Use of illegal drugs ▸ Risky sexual behavior ▸ Previous reception of tissue/organ transplant ▸ Recent (<12 months) reception of blood products ▸ Recent (<6 months) needle stick accident ▸ Recent (<6 months) body tattoo, piercing, earring, acupuncture ▸ Recent medical treatment in poorly hygienic conditions ▸ Risk of transmission of prions diseases ▸ Recent parasitosis or infection from rotavirus, *Giardia lamblia*, and other microbes with GI involvement ▸ Recent (<6 months) travel in tropical countries, countries at high risk of communicable diseases or traveller's diarrhea ▸ Recent (<6 months) history of vaccination with a live attenuated virus, if there is a possible risk of transmission ▸ Healthcare workers (to exclude the risk of transmission of multidrug-resistant organisms) ▸ Individual working with animals (to exclude the risk of transmission of zoonotic infections)	▸ History of inflammatory bowel syndrome or disease, functional chronic constipation, coeliac disease, other chronic GI disorders ▸ History of chronic, systemic autoimmune diseases with GI involvement ▸ History of, or high risk for, GI cancer or polyposis ▸ Recent appearance of diarrhea, hematochezia ▸ History of neurological/neurodegenerative diseases ▸ History of psychiatric conditions determining mental health instability or incapacity ▸ Overweight and obesity (body mass index >25)	▸ Recent (<3 months) exposure to antibiotics, immunosuppressants, chemotherapy ▸ Chronic therapy with proton pump inhibitors
**ISSUES TO ADDRESS ON THE SAME DAY OF DONATION TO CHECK ANY RECENTLY ONSET OF HARMFUL EVENTS**		
The following issues, if present, contraindicate donation on the same day on which they are assessed: ▸ Newly appeared GI signs and symptoms, for example, diarrhea, nausea, vomiting, abdominal pain, jaundice ▸ Newly appeared illness or general signs as fever, throat pain, swollen lymph nodes ▸ Use of antibiotics or other drugs that may impair gut microbiota, new sexual partners, or travels abroad since the last screening ▸ Recent ingestion of a substance that may result harmful for the recipients ▸ Travel in tropical areas—contact with human blood (sting, wound, showing, piercings, tattoos)—sexual high-risk behavior ▸ Diarrhea (more than three loose or liquid stools per day) among members of the entourage (including children) of the donor		
**BLOOD AND STOOL TESTING TO CHECK DONORS FOR ANY POTENTIALLY TRANSMITTABLE DISEASE**		
**General blood testing**	**General stool testing**	
▸ Cytomegalovirus ▸ Epstein-Barr virus ▸ Hepatitis A ▸ HBV ▸ HCV ▸ Hepatitis E virus ▸ Syphilis ▸ HIV-1 and HIV-2 ▸*Entamoeba histolytica* ▸ Complete blood cell count with differential ▸ C-reactive protein and erythrocyte sedimentation rate ▸ Albumin ▸ Creatinine and electrolytes ▸ Aminotransferases, bilirubin, gamma-glutamyltransferase, alkaline phosphatase	▸ Detection of *Clostridium difficile* ▸ Detection of enteric pathogens, including *Salmonella, Shigella* ▸*Campylobacter, Escherichia coli* O157 H7, *Yersinia*, vancomycin-resistant enterococci, methicillin-resistant *Staphylococcus aureus*, Gram-negative multidrug-resistant bacteria ▸ Norovirus ▸ Antigens and/or acid fast staining for *Giardia lamblia* and *Criptosporidium parvum* ▸ Protozoa (including *Blastocystis hominis*) and helminths ▸ Fecal occult blood testing	
**Blood testing in specific situations**	**Stool testing in specific situations**	
▸ Human T-lymphotropic virus types I and II antibodies *Strongyloides stercoralis*	▸ Detection of *Vibrio cholera* and *Listeria monocytogenes* ▸ Antigens and/or acid fast staining for *Isospora* and *Microsporidia* ▸ Calprotectin ▸*Helicobacter pylori* fecal antigen ▸ Rotavirus	

Analysis of T cell sub-populations will be performed both in peripheral blood and gut mucosa: Treg, Th17 cells, effector, and central memory cells.

At the end of the treatment period, further 6 months will be required as a follow-up period. This time is intended to assess late adverse events (AEs) and later biological or clinical effects of transplantation.

### Sample Size Estimation

Sample size was calculated considering as primary outcome measure the proportion of patients, in the transplantation group with respect to controls, displaying a “positive response” intended as an increase in the proportion of Treg by at least 20%. The null hypothesis states that FMT does not ameliorate significantly the proportion of positive responses in treated patients after the second FMT round, compared to their baseline and to control group. The alternative hypothesis is that FMT gives a proportion of positive responses in at least 50% of treated patients compared to a maximum 5% of positive responses in patients of the control group. The study was designed to refuse the null hypothesis with an alpha error of 0.05 and a power of 0.80 according to previously published statistical methods (26) and to known levels of Tregs in ALS patients, that typically display a slight reduction of Treg % (mean ± standard deviation: 2.1 ± 0.7) with respect to healthy controls with fast progressors patients having on average 31% fewer Treg (8, 27).

For this purpose, a population of 39 patients randomized in two treatment arms would suffice; taking into account an average drop out of 10%, a recruitment of 42 patients will be needed.

### Outcome Measures

Primary outcome measure is the modification from baseline to month 6 in Treg number in transplanted ALS patients compared to the control arm (26).

Secondary outcome measures include the following:

✓ Biological outcome measures:
  –Change from baseline to month 3, 6, 9, 12 of the T cell distribution especially the ratio Treg/Th1 or Treg/Th17 comparing FMT arm and control arm (both blood—at each time point, and small intestine—only at 6 and 12).
  –Changes from baseline to month 3, 6, 9, 12 in inflammatory status (cytokine profile) comparing FMT and control arm (in blood and feces at each time point, and in CSF at month 6).
  –Changes from baseline to month 3, 6, 9, 12 between FMT arm and control arm in the following biomarkers: creatinine, albumin, CK, vitamin D, plasma/CSF neurofilament heavy/light chain protein.
  –Biological modifications from baseline to month 6 and 12 in the composition of saliva, gut and fecal microbiota (including SCFA) comparing FMT and control arm.
✓ Safety: will be assessed in FMT and control arm considering the occurrence of AEs and modifications in physical examination, vital signs, body weight, and laboratory tests (biochemistry, hematology) during and following the treatment.
✓ Clinical outcome measures:
  The following clinical variables will be compared between FMT and control arm:
  – Change from baseline to each time point (month 1, 3, 6, 9, 12) of ALSFRS-R.
  – Survival from onset and randomization to death or tracheotomy.
  – Change of Forced Vital Capacity (FVC) from baseline to each time point (month 1, 3, 6, 9, 12).
  – Frequency of procedures (PEG, NIV, IV) from baseline to month 3, 6, 9, 12.
✓ Quality of life: will be assessed as the change in absolute and relative values of the Amyotrophic Lateral Sclerosis Specific Assessment Questionnaire (ALSAQ40) from baseline to month 6 and 12 in FMT and control arm.

The Table 3 shows the study flow chart.

**Table 3:** Study flow chart.

	**Pre-treatment**		**Treatment**					**Follow up**			**Study end**
**Examinations**	**Screening**	**Baseline (W0)**	**FMT 1 (Rome)**	**M1**	**M3**	**M6**	**FMT 2 (Rome)**	**M7**	**M9**	**M12 (Rome)**	**M12**
**Time window**		** <3 weeks from screening**	** <4 weeks from screening**	**±3 days**	**±3 days**	**±3 days**	**±7 days**	**±7 days**	**±7 days**	**±7 days**	**≥7 days <4 weeks from M12**
Informed consent	x										
Medical history	x										
Inclusion exclusion criteria	x										
Patient able to understand and follow procedures	x										
FMT			x				x				
**CLINICAL ASSESSMENT**											
Neurological examination	x	x		x	x	x		x	x		x
ALSFRS-R	x	x		x	x	x		x	x		x
FVC	x	x		x	x	x		x	x		x
MRC	x	x		x	x	x		x	x		x
BMI	x	x	x	x	x	x	x	x	x	x	x
**SAFETY ASSESSMENT**											
Adverse events		x		x	x	x		x	x		x
Vital signs	x	x	x	x	x	x	x	x	x	x	x
Physical examination	x	x	x	x	x	x	x	x	x	x	x
Concomitant medications	x	x	x	x	x	x	x	x	x	x	x
Chest X-ray	x (1)										
ECG	x (1)										
Hematology	x	x		x	x	x			x		x
Biochemistry	x	x		x	x	x			x		x
Urinalysis	x	x		x	x	x			x		x
Pregnancy test	x										
Infectious markers	x										
Fecal calprotectin			x				x			x	
**BIOLOGICAL ACTIVITY**											
Treg		x	x		x		x		x	x	x
Lymphocytes phenotype		x	x				x			x	
Fecal and saliva samples (microbiota)			x				x			x	
Gut tissue			x				x			x	
CSF		x				x					
Peripheral biomarkers		x			x	x			x		x
**QUALITY OF LIFE ASSESSMENT**											
ALSAQ40		x				x					x

*(1) If not done at diagnosis or in the last 12 months*.

*FMT, Fecal Microbial Transplantation; M, Month; MRC, Medical Research Council; FVC, Forced Vital Capacity; BMI, Body Mass Index; ALSFRS-R, Revised ALS Functional Rating Scale; ECG, Electrocardiogram; CSF, Cerebrospinal Fluid; ALSAQ40, ALS Specific Assessment Questionnaire; W, week.*.

### Adverse Events and Safety

According to Directive 2004/23/EC (implemented in Italy with D.Lgs 191/2007):

– “Serious adverse event” (SAE) means any untoward occurrence associated with the procurement, testing, processing, storage, and distribution of tissues and cells that might lead to the transmission of a communicable disease, to death or life-threatening, disabling, or incapacitating conditions for patients or which might result in, or prolong, hospitalization, or morbidity (https://www.hta.gov.uk/policies/human-application-adverse-event-and-reaction-saears-reporting; http://www.sc-toolkit.ac.uk/displaycontent.cfm?widCall1=customWidgets.content_view_1&cit_id=84).
– “Serious adverse reaction” (SAR) means an unintended response, including a communicable disease, in the donor or in the recipient associated with the procurement or human application of tissues and cells that is fatal, life-threatening, disabling, incapacitating, or which results in, or prolongs, hospitalization or morbidity (https://www.hta.gov.uk/policies/human-application-adverse-event-and-reaction-saears-reporting; http://www.sc-toolkit.ac.uk/displaycontent.cfm?widCall1=customWidgets.content_view_1&cit_id=84).

The existing studies suggest that FMT is a safe therapy, with few serious adverse events reported. There have been individual reports of peripheral neuropathy, Sjogren syndrome, idiopathic thrombocytopenic purpura, microscopic colitis, contact dermatitis, rheumatoid arthritis, obesity, bacteremia, and ulcerative colitis flare after FMT (24). In one meta-analysis, the overall incidence rate of adverse events after FMT was 28.5%. The commonest FMT-attributable adverse event was abdominal discomfort; also diarrhea, transient fever, nausea, vomiting, and constipation were other common symptoms associated to FMT (24, 28–31).

For these mild adverse events, investigators will treat symptoms according to the usual clinical practice. In case of symptoms that could be considered severe or life threating, the investigator will have to inform the sponsor immediately (within 24 h by fax using a SAE form) and second FMT round may be avoided accordingly to the local investigator's judgment. In accordance with the legislative requirements, Centers will be requested to report immediately from their awareness any SAE or SAR occurring during the trial and coordinating center will send Completed forms by email to Italian National Transplantation Center (CNT).

The coordinating Center will be responsible for appropriate AE reporting to the regulatory authorities (CNT) every 6 months; investigators will be responsible for reporting to appropriate Ethic Committee. In case of death, a clinical report will be prepared by the caring investigator together with SAE form; in case of autopsy, autopsy report will be added to study documentation.

The trial will be stopped in case of 30% excess in the treating group of the following AEs: peritonitis, upper gastrointestinal hemorrhage, sepsis, bacteremia. Instead, the next toxicities will be considered acceptable: diarrhea, abdominal cramping and pain, nausea, vomiting, flatulence, fever, constipation, dizziness, sore throat, rhinorrhea, bloating, nasal stuffiness, urinary infections, headache, mild/moderate blood cells alterations; elevated CRP, rashes, urticaria, dermatoses, dermatitis (mild/moderate severity), elevated aminotransferase/alkaline phosphatase, loss of appetite, colitis/gastroenteritis.

### Data Recording and Data Monitoring

An electronic case report form (CRF) will be prepared for data recording.

A certified contract research organization (CRO) will be in charge for monitoring the study.

The study monitor indicated by the coordinating center will be in contact with the investigators and will conduct a visit to each Center to discuss and/or collect data. The Monitor will conduct a visit before the start of the study to discuss the protocol and obligations of the investigators and sponsor. Investigators of each center are required to allow the Monitor to conduct the site visit, the study-end visit and the site closure visit.

The Investigators will make all pertinent records available including original medical documents for inspection by regulatory authorities.

Copies of the protocol, subject identification codes, electronic Case Report Form, source data, Informed Consent Form and other documents related to the study conduction and support the data collected from each subject will be stored for the maximum period of time as required by the study centers. No study document should be destroyed.

Originals of all documentation and copies of outgoing correspondence concerning the study will be stored and retained by the Sponsor in a safe area in the Trial Master File.

### Role of Participating Centers

This multicenter study will involve nine Italian Units: six referral ALS Centers (located in Florence, Chieti, Perugia, Modena, and Rome), three renowned laboratories, one endoscopy service and a statistical unit.

ALS is a rare disease with fast progression and no effective treatment, which requires a solid methodological, clinical approach, and biological background for clinical trials conduction.

Each clinical center is expected: (I) to randomize at least seven patients according to including and excluding criteria in 18 months; (II) to provide one principal investigator to evaluate including and excluding criteria, and assess primary and secondary outcomes; (III) to adhere to ALS management guidelines of the European Federation of Neurological Societies(in particular as regards ventilation and nutrition issues).

The analysis of biomarkers will be centralized and performed in two internationally renowned laboratories Laboratory of Microbiology Policlinico Gemelli in Rome and Laboratory of Immunology, Department of Experimental and Clinical Medicine—University of Florence, and FMT will be performed at Policlinico Gemelli in Rome, a leading European Center for this kind of treatment.

### Data Analysis

Data will be collected by investigators through electronic CRF, which will be conveyed to the trial database. At trial completion, data from the locked database will be extracted for analysis by an expert statistician.

Separate analyses will be performed in:

1. All randomized subjects receiving at least 1 FMT (Intention-to-treat population).
2. All randomized subjects excluding protocol deviations (Per protocol population).

As far as biological activity is concerned, immune response to FMT will be analyzed as a difference in positive response to FMT between the control group and the FMT group, assessed by comparison of proportion of patients in the two groups showing a mean Tregs increase by at least 20%, between baseline and month 6 measurements.

Mean values of different T, B, NK cell subpopulations, neurodegeneration biomarkers, and cytokines will be assessed and mean differences in plasma concentrations between the two treatment arms will be calculated using *t*-test or Wilcoxon-Mann-Whitney test as previously reported (26). ANOVA will be used for assessing the mean change over time for the same variables as above, with treatment as between-subjects factor and time as within-subjects factor. Different models will be used, each with a different biomarker of activity as the dependent variable. Models will be adjusted for any unbalanced distribution of the main prognostic factors (e.g., age) between the two treatment arms, according to previously published statistical methods (26).

Analysis of microbiota will be performed with specific software.

Record of any AE and SAE will be kept for every subject receiving at least one round of FMT till the study completion, performing safety analysis accordingly.

As for clinical outcome measures, we will compare ALSFRS-R total score and subscores (bulbar, respiratory, gross, and fine motor) changes from baseline to each time point in treatment arm vs. control arm. Frequency of procedures (PEG, NIV, IV) will be compared from baseline to each time point in treatment vs. control arm. The comparison of clinical endpoint among arms will be carried out by using the logistic regression model. Results will be presented as odds ratios (OR) and 95%C.I. FMT will be considered effective in relation to controls whether the OR of positive results will provide a *p* < 0.05. Change in FVC during the study will be analyzed using a mixed model for repeated measures (MMRM). Difference between treatment groups and two-sided 95% CI will be estimated .Log-rank tests via Kaplan-Meier method will be employed to compare differences in tracheostomy-free survival between the two treatment arms (from onset and from randomization), while Cox's proportional hazard model will be used for adjusting for any possible unbalanced prognostic factors. Statistical significance will be set at 0.05 level for a two-tailed test. Last observation will be considered for patients with missing data.

Given the short duration of the study, an interim analysis of efficacy data is not scheduled. Nevertheless, to address safety concerns, a report including all relevant clinical data of patients will be sent by the Statistics Unit to an independent Data and Safety Monitoring Board for scheduled DSMB meetings (also through Skype) when 14 patients would have completed the second treatment and then every 6 months.

### Ethics Approval

This study has been approved by Comitato Etico Area Vasta Emilia Nord on July 2018 (prot. n. 0010722/18), and by National Transplantation Center on 29 March 2019.

The trials has been registered in clinicaltrials.gov (NCT03766321).

## Discussion

### Choice of Treatment

FMT dates back to fourth-century China, as a report of a traditional Chinese medicine doctor describing a patient's recovery from food poisoning and severe diarrhea after treatment with oral human fecal suspension (32). Further uses of this kind of treatment have been described since the sixteenth century (32–35) mainly through retention enema.

More recently, FMT has emerged as a safe and effective treatment for the management of recurrent, and possibly refractory, *Clostridium Difficile* Infection (CDI) by restoring gut microbial diversity, with cure rates >85% (36). Currently, the FMT has been approved for the treatment of this condition, for which it has had unanimously excellent results (24, 37, 38).

Since a perturbed microbiota is associated with several diseases, it is conceivable that microbiota restoration therapies could be useful in their management (24). The importance of “healthy” gut microbiome has been shown in oncology, an in detail in relation to response to antitumor treatment (39–41). In addition, a few papers have recently highlighted a potential link of gut dysbiosis and neurological diseases (41–45), such as multiple sclerosis (46, 47), Parkinson's Disease (48, 49), and Alzheimer's disease (50, 51).

For this reason, there are studies now testing FMT potentiality to treat multiple sclerosis, myasthenia gravis, Parkinson's Disease, and epilepsy (22, 52).

Some preliminary data place the theoretical basis for a GM involvement in ALS because immune system, which plays a key role at least in ALS progression, can be modulated through gut-brain axis. Indeed, the gut environment favors the generation of autoreactive T-cells with unique regulatory functions, important for preventing CNS autoimmunity (53). Some commensal bacteria can induce Tregs development and FMT determine Tregs' increase (54).

Moreover, alterations in GM composition in ALS have been reported in previous studies. Fang et al. (55) found a significantly increased population of harmful microorganisms (genus *Dorea*) with reduced population of beneficial microorganisms (genus *Oscillibacter, Anaerostipes, Lachnospiraceae*) in ALS patients (45). The authors suggested that the imbalance in intestinal microflora constitution may cause a pro-inflammatory dysbiosis that may alter the intestinal epithelial barrier, promoting immune/inflammatory responses with a major role in ALS pathogenesis.

Another study (56) detected a higher amount of *E. Coli* and *Enterobacteria* and a low presence of total yeast in the GM composition of ALS patients with respect to healthy controls.

Two pre-clinical researches, performed in G93A animals, studied the correlation between gut dysbiosis, altered intestinal permeability and enteric inflammatory/neurogenic responses (12). Wu et al. (57) found signs of leaky intestine in a G93A transgenic mouse having an augmented gut permeability due to impairment of the intestinal tight junction structure and related protein expression, if compared to wild-type mice (57). The same model showed a reduced number of and an altered function of epithelial Paneth cells, that impact the GM and have a role in the innate immune response, and, as far as concerns GM composition, a lower abundance of butyrate-producing bacteria such as *Butyrivibrio fibrisolvens, Escherichia coli*, and *Firmicutes* was detected. Finally, shifts in the gut microbiome included.

Likewise, Zhang et al. (58) reported a correlation between gut dysbiosis and morphofunctional alterations of intestinal permeability in the same model, since the earliest stages of the disease. They demonstrated the presence of SOD1 aggregates, which are distinctive of ALS associated to SOD1 mutations in animal models and in patients, not only in neurons and skeletal muscle, but also in the intestine of ALS mice and human intestinal epithelial cells. Moreover, the authors observed that, following treatment with 2% butyrate (a natural bacterial product able to restore the intestinal microbial homeostasis), G93A mice restored GM balance and intestinal epithelial barrier integrity, besides they showed improved central and peripheral symptoms of the disease, prolonged survival, and slowing of weight loss (58).

These data suggested that changes in GM, impaired intestinal permeability and enteric inflammation represent one of the earliest events in ALS. However, these findings did not allow to firmly establish whether the alterations of the enteric bacteria neuro-immune network contribute to the ALS pathophysiology, or whether they happen as a consequence of the cascade of events accompanying neurodegeneration (12).

In a recent study, SOD1-Tg mice prone to ALS showed a vivarium-dependent pre-symptomatic dysbiosis and an altered configuration of metabolites, occurring with a disease worsened under conditions of germ-free or broad-spectrum antibiotic treatment (59). The authors could correlate some species in gut microbiota with ALS severity and supplementation with certain species changed mice phenotype (59).

In this context, FMT may act against ALS progression, by regulation of the mutual signaling between gut microflora and CNS (11), employing bidirectional communication (via neuronal, hormonal, immunologic, and toxic signaling) (14, 15). Moreover, direct communication through the vagus nerve, changes in tryptophan and norepinephrine metabolism, production and absorption of neuroactive metabolites, immune activation through molecular mimicry and the direct production of neurotoxins (22) may be useful in controlling ALS disease) (13). Nearly 30% of ALS patients show autonomic dysfunctions with demonstrated involvement of the intermediolateral columns and the Onuf nucleus. The vagal nerve could be a route for GM and brain communication (60). Of note, GM has been found to interact with ENS-vagus nerve pathways (61) because, bacterial derived-neurotransmitters and neuropeptides can activate directly myenteric neurons, which, through vagal nerve ascending fibers, deliver nerve inputs to the brain (12, 62).

Enteric bacteria and their metabolites (especially the SCFAs) can indeed induce enterochromaffin cells to release neurotransmitters and neuropeptides (including peptide YY, neuropeptide Y, cholecystokinin, glucagon-like peptide-1 and−2, and substance P), which, in turn, can reach the brain through blood circulation and have an effect on CNS functions (12). In addition, the intestinal epithelium regulates the spread of specific bacterial products (e.g., SCFAs, vitamins or neurotransmitters, such as acetylcholine, dopamine, noradrenaline, gamma-aminobutyric acid, or serotonin) into the circulatory system, that, in turn, may arrive to the CNS (11, 12). In this way, circulating microbiota-derived metabolites, neuropeptides, and neurotransmitters can enter the CNS and so directly influence the neurobiology and the ALS pathology.

Individual case reports of ALS patients documented benefits as a result of FMT (63). This research field is therefore highly innovative and is gathering increasing interest from the international scientific community (13, 43).

### Anticipated Results

Our trial aims at evaluating the biological bases of FMT as a potential treatment for ALS in order to plan a following efficacy study on fecal microbiota transplantation for this disease.

We will carry on the first clinical trial with FMT in ALS patients; this is a highly innovative therapeutic approach that will give information about safety and tolerability of FMT. We will assess biological FMT effects in ALS and we will have some preliminary clinical data about the possibility that microbiota-based treatment approaches can represent a new therapeutic target for ALS.

The strength of our study is represented by a large panel of biological tests aimed at clarifying the role of enteric bacteria-neuro-immune network in ALS patients, since the earlier stages of the disease (patients will be enrolled only if disease onset <18 months), through exploration of immunological patterns in blood, CSF, saliva, and feces, along with small intestine tissue in ALS patients treated or not with FMT. These data will be correlated with composition of saliva, gut and fecal microbiota at different time points, allowing to contribute to the understanding of ALS pathophysiology in terms of microbiota involvement in the causal ALS process, or as a consequence of the central neurodegenerative processes.

Moreover, comprehensive patients' clinical phenotyping will be accomplished according to the study protocol, allowing to establish correlations between precise groups of progressors and the GM system, which has never been explored in terms of biomarker of disease progression. Further analyses considering subgroups of patients on the basis of Treg cells or microbiota population will help to better understand underlying pathomechanisms of the disease and to plan more targeted advanced studies.

The sampling from different matrices will grant a full view of the modifications operated by FMT and in the future, more accessible biomatrices such as saliva might be considered for monitoring disease status in ALS patients. This trial has the potential to give preliminary results to carry on further larger studies aimed at assessing FMT effectiveness in ALS treatment.

## Ethics Statement

This RCT will be performed in compliance with the Declaration of Helsinki, as amended by the 64th WMA General Assembly, Fortaleza, Brazil, October 2013, and with the current ICHGCP-guidelines.

The study has been approved by lead ethics committee (Comitato Etico Area Vasta Emilia Nord on July 2018) and has been submitted to local ethics committees. The study obtained the approval of National Transplantation Center and Superior Council of Health (as competent authorities for experimental transplantation) on March 2019.

All subjects, after comprehensive written and verbal information, will date and sign an approved Informed Consent Form (ICF) explaining rationale, procedures, duration, possible risks and benefits associated with the study. The patient will be informed that participation in the study is voluntary and that refusal to participate or withdrawal from the study, at any time, will not be associated to any penalty, or loss of benefits. An insurance company will provide insurance coverage for damages to patients involved in the trial. Principal Investigator will be supplied with all data concerning the insurance company and policy number. Finally, the data privacy and confidentiality will be treated according to European and Italian law. An Independent Ethics Advisory Board (IEAB) has been established, to address ethics concerns that could arise during the study, focusing especially to informed consent form. For this reason patient information sheet will be given to the candidate patient at least 15 days before the collection of the ICF. In the patient information sheet the patient will find a telephone number of the IEAB that he/she could call to have independent information. Furthermore, when the neurologist will give the patient information sheet to the patient, the investigator will advise IEAB of a possible enrollment to allow IEAB member to be present at the moment of the ICF collection. An independent Data Safety and Monitoring Board (DSMB) has been established too, to address safety and efficacy concerns that could arise during the study. Reports including all relevant clinical data of patients will be sent by the Statistics Unit of the University of Modena to the independent DSMB for scheduled DSMB meetings when 14 patients have done the second treatment and subsequently after every 6 months. All the partners will guarantee the dissemination and exploitation of the scientific results within the consortium and externally (international conferences, publications, dedicated workshops with patients). The results will be presented during national and international neurological and ALS meetings and workshops. Results will be published on www.clinicaltrials.gov and in peer-reviewed international journals.

## Author Contributions

AA, JM, and LM contributed to conception and design of the study. RD'A contributed to design of the study and planned statistical analysis. EZ and JM wrote the first draft of the manuscript. AA, EN, EZ, GC, LM, RD'A, and TS wrote sections of the manuscript. All authors contributed to manuscript revision, read, and approved the submitted version.

### Conflict of Interest

The authors declare that the research was conducted in the absence of any commercial or financial relationships that could be construed as a potential conflict of interest.

## FETRALS Working Group

Neurology Unit, S. Agostino Estense Hospital, Department of Neuroscience, Azienda Ospedaliero Universitaria di Modena, Modena, Italy; Department of Biomedical, Metabolic and Neural Sciences, University of Modena and Reggio Emilia, Modena, Italy: Jessica Mandrioli, Nicola Fini, Elisabetta Zucchi, Annalisa Gessani, Antonio Fasano, Ilaria Martinelli.

Laboratory of Immunology, Department of Experimental and Clinical Medicine, University of Florence, Florence, Italy: Amedeo Amedei, Elena Niccolai, Federica Ricci.

Scienze gastroenterologiche, endocrino-metaboliche e nefro-urologiche, Fondazione Policlinico Universitario A. Gemelli IRCCS, Università Cattolica del Sacro Cuore, Rome, Italy; Istituto di Medicina Interna e Geriatria, Università Cattolica del Sacro Cuore, Rome, Italy: Giovanni Cammarota, Antonio Gasbarrini.

Instituto di Microbiologia, Università Cattolica del Sacro Cuore, Rome, Italy Dipartimento di Scienze di Laboratorio ed Infettivologiche, Fondazione Policlinico Universitario A. Gemelli IRCCS, Rome, Italy—Luca Masucci, Teresa Spanu, Gianluca Quaranta, Maurizio Sanguinetti.

Statistics Unit, Department of Medical and Surgical Sciences for Children & Adults, University of Modena and Reggio Emilia, Modena, Italy: Roberto D'Amico, Roberto Vicini.

Neuromuscular Omnicenter (NEMO), Fondazione Serena Onlus—Fondazione Policlinico A. Gemelli, Rome, Italy, & Institute of Neurology, Catholic University of the Sacred Heart, Fondazione Policlinico A. Gemelli, Rome, Italy: Mario Sabatelli, Amelia Conte.

Institute of Neurology, Policlinico Campus Bio-Medico, Rome, Italy: Vincenzo di Lazzaro.

Department of Neurological and Psychiatric sciences (NEUROFARBA), University of Florence, Florence, Italy, & Careggi University Hospital, Neurology unit, Florence, Italy: Sabrina Mata, Sandro Sorbi.

Neurology Clinic, “S Annunziata” Hospital, Chieti, Italy, & Department of Neuroscience and Imaging, University “G. d'Annunzio” of Chieti-Pescara, Chieti, Italy: Margherita Capasso, Marco Onofrj.

Perugia Hospital, Neurophysiopathology Unit, Azienda Ospedaliera di Perugia, S. Andrea delle Fratte, 06156 Perugia, Italy: Teresa Anna Cantisani, Paola Brunori.
